# Advances in Organoid Research and Developmental Engineering

**DOI:** 10.3390/bioengineering11121275

**Published:** 2024-12-15

**Authors:** Peter V. Hauser

**Affiliations:** 1 Department of Medicine, David Geffen School of Medicine, University of California Los Angeles, Los Angeles, CA 90095, USA; pvhauser@ucla.edu; 2 Medical and Research Services, Greater Los Angeles Veterans Affairs Healthcare System at Sepulveda, North Hills, CA 91344, USA

Over the last decade, organoid research has emerged as a transformative field in biomedical science, offering unparalleled opportunities in disease modeling, pharmacological testing, and tissue engineering. The number of research articles published in the field has increased exponentially from 189 in 2014 to 3899 in 2024. This Special Issue of *Bioengineering* highlights studies showcasing advances in organoid research.

The Special Issue opens with a comprehensive review of organoid imaging technologies by Fei et al., emphasizing state-of-the-art approaches like light-sheet microscopy and multi-photon imaging for tracking organoid development at a cellular resolution [1]. The authors suggest that in order to enhance organoid-based drug discovery and disease modeling efforts, integrated imaging platforms for whole-object 3D imaging are necessary to enable non-invasive organoid monitoring and evaluation.

The effects of α2-adrenergic agonist brimonidine in glaucoma disease modeling are the subject of two studies by the Ohguro group. Watanabe et al. describe how the α2-adrenergic agonist brimonidine mitigates TGF-β2-induced fibrosis-related changes in a 2D and 3D in vitro model that replicates the effect of glaucoma on the human trabecular network [2]. It appears that brimonidine reduces the barrier function in trabecular cell monolayers and reduces the stiffness in 3D spheroids. On the other hand, Umetsu et al. demonstrate that brimonidine significantly modulates the adipogenic effects induced by ROCK inhibitors, particularly ripasudil, by suppressing lipid accumulation and changes in extracellular matrix components in differentiated adipocytes [3]. Both studies suggest a potential therapeutic application of brimonidine in glaucoma treatment. The quest to find chemical cues that drive retinoic acid-induced remodeling in human conjunctival fibroblasts is addressed by Tsugeno et al. [4]. Their findings elucidate how retinoic acid influences extracellular matrix dynamics in 2D and 3D cultured human conjunctival fibroblasts, offering critical insights for tissue regeneration strategies.

Karakaidos et al. compare the genotoxic effects of UV- and visible light-based bioprinting [5]. By assessing DNA damage, the authors propose bioprinting protocols that balance cell viability with reduced genotoxic risks, advancing tissue engineering applications. An interesting study by Sánchez-Salazar et al. presents a 3D-printed tumor-on-chip platform for colorectal cancer research [6]. They demonstrate how microsphere cultures in the chip enable precise drug testing while replicating in vivo-like environments, making strides toward more effective and personalized cancer treatments. In the realm of organ development, Hamon et al. explore how oxygen levels and the initial absence of tissue vascularization influence kidney organoid branching [7]. Their work highlights the critical role of hypoxia in modulating cellular subpopulations, providing a platform for understanding kidney morphogenesis and potential regenerative therapies. Finally, Hernandez et al. present a semi-3D bioprinted neurocardiac model that integrates cardiac and neuronal tissues [8]. Their innovative design demonstrates functional neuro-cardiac coupling, creating a robust platform for studying the cardiac autonomic nervous system under healthy and diseased conditions.

Collectively, these studies exemplify the innovative strides being made in organoid research. As organoid technologies advance, the integration of organoid models with integrated analysis platforms and artificial intelligence will be crucial for driving translational research and therapeutic applications. This Special Issue highlights efforts to shape the future of regenerative medicine, precision therapeutics, and organoid-based disease modeling.
